# Current Concepts in Probiotic Safety and Efficacy

**DOI:** 10.3390/nu18040696

**Published:** 2026-02-21

**Authors:** Alexey A. Churin, Ludmila O. Sokolyanskaya, Anastasia P. Lukina, Olga V. Karnachuk

**Affiliations:** 1 Laboratory of Microbial Technologies, Tomsk State University, Tomsk 634050, Russia; lusi5055@yandex.ru (L.O.S.); anastasiya-lukina-93@mail.ru (A.P.L.); 2 Department of Plant Physiology, Biotechnology, and Bioinformatics, Tomsk State University, Tomsk 634050, Russia; olga.karnachuk@green.tsu.ru

**Keywords:** probiotics, regulatory control, live biotherapeutic products, probiotic safety, lactobacilli, big data, artificial intelligence

## Abstract

**Background/Objectives**: Advances in molecular biology, genetics, and microbiome research have significantly expanded our understanding of probiotic microorganisms and their interactions with human health, stimulating the development of both traditional and next-generation probiotic products. Although probiotics are widely used and generally considered safe for healthy individuals, accumulating evidence indicates that their safety profile varies significantly depending on the strain, dose, host, and context, with rare but clinically significant adverse events reported in vulnerable populations. **Methods:** This review summarizes current knowledge on the efficacy and safety of probiotics, analyzes limitations in clinical safety reporting, and compares regulatory frameworks governing the use of probiotics as dietary supplements, medicinal products, and live biotherapeutics. Particular attention is given to new genomic and computational approaches to safety assessment. **Conclusions:** Overall, the review emphasizes the need for coordinated regulation, rigorous clinical evidence, and integrated, modern safety assessment strategies to support the responsible expansion of probiotic use.

## 1. Introduction

Fermented dairy and dairy products have accompanied human history for many millennia, from home production to large-scale industrial production at the modern stage of food industry development. Various fermentation technologies involving different strains of lactic acid bacteria are used to improve the consumer properties of the valuable products obtained [1]. Among the acquired properties, one can list extended shelf life, enhanced ability to inhibit the reproduction of pathogenic microorganisms, promotion of the efficient absorption of nutrients, and provision of a positive complex effect on the body due to the influence of lactic acid bacteria [2].

Mechnikov’s early 20th-century theory suggested that Bulgarian yogurt could extend life by suppressing harmful intestinal fermentation, a concept that gained wider medical support in the mid-1990s [3]. The intensive development of omics technologies and their application in microbiome research has significantly expanded the understanding of human microbiota, both as a whole and at the individual genomic level [4,5]. At the same time, researchers and developers are actively pursuing probiotics, which face several challenges, including safety assessment issues, the divergence of regulatory requirements, and their lag in technological development. The assessment of probiotic efficacy and its strain- and species-specificity is also challenging. These issues are particularly common with next-generation probiotics (NGPs), which lack a long safety history.

Owing to the unique mechanism of action of probiotics, their functions are much broader than commonly accepted. These functions include the production of metabolites necessary for the macroorganism, strengthening the protective barrier of the intestinal mucosa, modulation of the immune system’s state, and competition with pathogenic microorganisms [6,7]. Probiotic microorganisms can lower lipid concentrations in the blood, preventing and treating cardiovascular diseases, diabetes, and allergic diseases [8], regulating the state of the central nervous system through modulation of the “brain–gut” axis [9], performing detoxification functions [10], and exhibiting antimicrobial [11], antiviral, and antitumor properties [12]. The mechanism of action is determined by the ability of probiotics to act in three general modes: positive effect on barrier function, immune modulation, and metabolite production [13]. Simultaneously, an analysis of the results of clinical studies provides a contradictory picture of the confirmed effects of probiotics [14,15].

Probiotics currently available on the pharmaceutical market primarily belong to the following families, genera, and species: *Lactobacillaceae*, *Bifidobacterium*, *Streptococcus*, *Leuconostoc*, *Propionibacterium freudenreichii*, *Bacillus*, *Pediococcus* (QPS List (EU) or GRAS (FDA)—confirm species-level history of safe use) and less commonly used *Enterococcus faecium* (QPS status with qualifications) and *Saccharomyces cerevisiae* (QPS List (EU)/GRAS (FDA)) [16,17]. They belong to the group of so-called traditional probiotics [10]. Among them, lactic acid bacteria, particularly *Bifidobacterium* and *Lactobacillaceae*, are the most widely distributed phylogenetic groups [8].

It has been shown that to achieve a pronounced positive health effect, a daily dose of probiotics must reach levels of 10^8^–10^11^ CFU [13]. Lower doses are generally ineffective due to the death of most microbes in the acidic environment of the stomach [7]. For example, the survival rate of bacteria during passage through the stomach and interaction with bile salts ranges from 20% to 40% [18], and of the 10^8^ CFU of orally ingested lactobacilli, only 10^7^ are found in the intestines [7]. As noted in the scientific literature, this is an important safety factor for probiotics, especially in vulnerable populations, along with dose-dependent effects, translocation, antibiotic resistance, and special host-related problems [19,20].

With the spread of new technologies and methods for studying the human microbiome and the expansion of knowledge about its significance for health, researchers have recently turned their attention to so-called NGPs (Table 1).

Unlike traditional probiotics, which are primarily aimed at improving gut health, NGPs are specifically developed for the treatment of specific diseases with targeted therapeutic effects [21]. Like traditional probiotics, NGPs are live microorganisms, whose identification has been carried out based on comparative microbiota analysis. When administered in adequate quantities, they are expected to benefit the host’s health, providing more specific effects [22]. Typically, these are specially developed species that have never been used before in the food or pharmaceutical industries and therefore raise significant safety concerns (genomic stability, host-specific risks, etc.) due to regulatory uncertainty, which will be discussed in the following sections.

The development of such probiotics is usually conducted in comparison with the microbiota of sick and healthy individuals, and information regarding their safety is either incomplete or absent. The screening strategy for NGPs is similar to that used in the development of new drugs, aimed at identifying the targets and mechanisms of efficacy of the biopharmaceutical product for which this group of probiotics is being developed [23].

The most promising NGPs have been identified among *Bacteroides*, *Clostridium*, *Faecalibacterium*, *Eubacterium*, and *Akkermansia* [10,16]. The list of candidates continues to expand due to active ongoing research and NGPs are attracting increasing attention from a wide range of developers, as evidenced by the constantly growing volume of publications on research results.

In addition to the creation of products based on live microorganisms, other approaches are being developed to create products with probiotic activity without using live bacteria, which are associated with certain challenges, such as standardization or maintaining beneficial properties from production to end-user application.

For example, prebiotics are products that support the health of the host’s microbiota through modulatory properties but contain non-living food ingredients [4]. The term “synbiotics” is used to refer to products that include both probiotics and prebiotics, in which the beneficial properties of bacteria are supported by components that stimulate the growth of these same bacteria (Table 1) [24].

The definition of a product group under the general name “postbiotics” is still under discussion. The term “postbiotics” is increasingly found in scientific literature and on commercial products, but is used inconsistently and, until recently, has not been clearly defined [25]. However, similar to the previous groups, they are intended to have a beneficial effect on the host’s body and contain substances released because of the metabolic activity of microorganisms [7]. A factor that increases interest in postbiotics is their important characteristic of stability during industrial production and storage [25,26]. The use of dead cells in postbiotics eliminates the problem of fluctuations in quantitative composition during shelf life and is a good alternative to other groups [25,27]. Probiotics are considered safe due to their composition and may also be suitable for most vulnerable populations, such as infants, the elderly, and those with weakened immune systems [28].

The lack of standardized methods for assessing dose–response relationships, characterization and quality control of postbiotic products and their production limits their wider acceptance and use. The development of reliable quality control measures for postbiotics, validated methods for analyzing active ingredients, and harmonization of international standards will help address these gaps [26].

The scientific literature suggests that developing prebiotics and postbiotics is more promising due to the more accessible control and standardization for these products (Table 1) [4]. Meanwhile, products based on live microorganisms are characterized by a complex regulatory system and are registered as medicinal products. These systems are still developing, even at the international level.

This review comprehensively examines modern concepts of probiotic safety and efficacy, highlighting regulatory frameworks and the need for their harmonization, challenges in assessing therapeutic efficacy based on clinical trial data, current approaches to studying probiotic activity and safety using modern computational methods, and barriers to the development of effective probiotics, including next-generation products.

## 2. Regulatory Framework for Probiotics

### 2.1. International Experience

Historically, there was no unified approach to evaluate the benefits and efficacy of live microorganisms because regulatory authorities had not developed clear procedures for their assessment. Developments were conducted in two main areas. Food products or dietary supplements based on live organisms for maintaining and restoring the intestinal microbiota to support health were the first direction. The second direction involved medicinal products with therapeutic activity for treating human diseases, containing live microbial strains and registered in the prescribed manner. Although both directions evolved within the framework of the concept of probiotics, the products were intended for different target groups. Food products, dietary supplements, and nutraceuticals are intended for use by healthy individuals, whereas medicinal products based on live microorganisms are designed for treating or preventing specific diseases. Therefore, the regulation of these areas of probiotic application differs significantly.

The regulation of probiotic products varies significantly depending on their intended use. Probiotics marketed as foods or dietary supplements are subject to food and consumer product safety legislation. In contrast, live biotherapeutic products, intended to treat or prevent diseases, are regulated as biological medicinal products. This means that they must undergo rigorous safety, efficacy, and quality testing similar to that applied to pharmaceuticals [29,30]. Key regulatory aspects include accurate strain identification and traceability, comprehensive safety assessment (including whole-genome analysis for potential virulence and antibiotic resistance), proven efficacy for the intended use, and robust manufacturing controls to ensure product stability and purity [31,32,33]. While food probiotics require proof of safe use or QPS status, LBPs require controlled clinical trials, detailed manufacturing and quality control documentation, and extensive genomic and pharmacovigilance data [20]. Therefore, early product classification is critical because it determines the required body of evidence and the regulatory pathway for marketing authorization and subsequent oversight.

Beyond procedural differences, regulatory classification shapes innovation directions and development strategies. Pharmaceutical-level regulation ensures safety and a rigorous evidence base, but significantly increases costs, timelines, and financial risks, especially for next-generation probiotics not previously used in clinical practice. Consequently, developers may prioritize developing dietary supplements, which provide a faster time to market but limit therapeutic claims. Thus, while strict classification protects public health, it can also limit flexibility in implementation and influence which innovations reach clinical application.

Currently, the generally accepted definition of “probiotics” is the formulation proposed by the Food and Agriculture Organization of the United Nations (FAO) in 2001, which states that “probiotic is a live microorganism that, when administered in adequate amounts, confers a health benefit on the host” [34].

Since the FAO definition did not establish clear boundaries for distinguishing probiotics for use as food or medicinal products, later terms such as “pharmabiotics” [35] or “live biotherapeutic products (LBP)” [36] were proposed (Table 2). The U.S. Food and Drug Administration (FDA) characterizes LBP as “a new therapeutic approach that includes live microbes (e.g., bacteria, yeast), is not a vaccine, and is used for the prevention, treatment, or cure of human diseases” [36]. Such a therapeutic product may include either a single microbial strain or a consortium of several microbial strains.

Later, in 2019, the concept of LBP was established in the European Pharmacopoeia (Ph. Eur.), where possible routes of administration (oral or vaginal) and various types of pharmaceutical forms were described in addition to defining the term LBP itself (Table 3) [37,38].

Notably, Europe has the strictest regulations and generally discourages any health claims made about probiotics (Table 3) [39]. This inevitably impacts the number of probiotics registered for medical use. Experts note that there is still no single, globally agreed-upon legal definition of “probiotic,” leading to consumer confusion and trade barriers. There is also a clear need for more specific regulations covering not only commercial products but also the various stages of clinical development, validation of the entire process, and commercialization [40].

Differences in legislation contribute to the structural heterogeneity of the global probiotics sector. Different definitions, thresholds for demonstrating efficacy, and acceptable claims require manufacturers to develop jurisdiction-specific documentation and labeling strategies, increasing costs and delaying commercialization. Inconsistent interpretations of the term “probiotic” further complicate scientific collaboration. Consequently, regulatory heterogeneity functions not simply as an administrative distinction but also as a bottleneck toward international harmonization and equitable market access.

Currently, pharmabiotics are being developed with the prospect of further registration in the United States across several areas, including gastrointestinal diseases (such as diarrhea caused by antibiotic or cancer treatment, constipation, irritable bowel syndrome, etc.), allergic diseases (such as rhinitis, dermatitis, asthma, etc.), dental diseases (such as caries, gum disease), and disorders related to the gut–brain axis (including stress, depression, autism spectrum disorders, schizophrenia, Alzheimer’s disease, and Parkinson’s disease) [41].

The classification of developed probiotics as medicinal products requires developers to provide evidence that the benefits of using the probiotic outweigh the potential risks. Such evidence of benefit and low risk of adverse effects results from multi-stage preclinical and clinical observations, conducted in compliance with the relevant regulatory requirements [42].

Risk categories for probiotics are associated with four interrelated factors: acquired antimicrobial resistance genes, virulence factors, translocation, and mobile or unstable genomic elements that allow horizontal gene transfer [43,44]. Taken together, these factors can increase the pathogenic potential or expand the host microbiota’s resistance. Regulatory assessment varies by product category: a history of safe use (or QPS status), compositional control, and manufacturing quality are of significant importance for food-grade probiotics, whereas assessment is conducted according to drug or biological standards requiring preclinical safety, controlled efficacy trials, compliance with GMP standards, and enhanced genomics and pharmacovigilance data for therapeutic LBPs [29,32].

These regulatory differences extend beyond administrative procedures and directly impact clinical standardization and practice. Variability in strain characteristics, the depth of genomic screening, antimicrobial resistance assessment, and manufacturing requirements reduces product comparability, despite similar labeling. Consequently, physicians may encounter probiotics supported by significantly different levels of evidence, complicating therapeutic decision-making and the development of recommendations. Thus, regulatory heterogeneity impacts not only market access but also clinical confidence and integration into evidence-based medicine.

The registration procedure for LBPs in the European Union falls under the requirements of Directive No. 2001/83/EC of the European Parliament and the Council of the EU “On the Community Code Relating to Medicinal Products for Human Use” (Table 4). This directive also outlines the requirements that the information and documents accompanying the application for marketing authorization must demonstrate that the therapeutic effectiveness of the product outweighs the potential risks. Since LBPs are intended for both the prevention and treatment of diseases, this group of probiotics is classified as medicinal products under the directive, requiring regulatory approval before being brought to market [45].

A major challenge is the lack of clarity in the regulatory framework for NGPs, which are specifically designed with enhanced properties, such as those obtained through synthetic biology, to provide a therapeutic effect [21,22]. NGPs, as well as biotherapeutic drugs (LBP) or medicinal products, must undergo pharmaceutical clinical trials and studies of their pharmacokinetics and pharmacodynamics with the wide use of new technologies such as genetic sequencing and bioinformatics [22].

In Europe, the European Medicines Agency (EMA) reviews applications for medicinal product registration, whereas the European Food Safety Authority assesses food products (EFSA) (Table 2). The dossier submission procedure is not sufficiently detailed in both cases [47]. The situation is complicated by the fact that probiotics are live microorganisms, requiring the development of additional methods for standardization and quality determination. For this purpose, for example, the use of flow cytometry to assess live, dead, and active microorganisms has been proposed and subsequently included in the European Pharmacopoeia (Ph. Eur.) in the corresponding article (Microbiological Examination of LBP: Test for the Presence of Specified Microorganisms (2.6.38)) [37].

According to the minimum criteria for probiotics established by the International Scientific Association for Probiotics and Prebiotics (ISAPP), in addition to general information about the manufacturer, storage conditions, intended use, administration methods, and dosage, the identification of microorganisms by genus and species for all strains in the product must be provided [48]. Moreover, the FDA recommends using at least two additional methods for microorganism identification [36].

According to the European Medicines Agency the product registration dossier should include the complete genome sequence of the strain, along with a detailed list of identified antibiotic resistance genes, multi-drug resistance clusters, and potential virulence factor genes. In the case of LBPs, the characterization of the strain(s) is also part of the safety documentation included in the registration dossier [36,49]. However, to date, there are no specific guidelines from EMA or FDA that provide detailed recommendations regarding the quality of genome sequencing and the associated bioinformatics analysis [50].

Given the still-developing regulatory requirements for the composition of registration dossiers for LBPs, strains and their combinations that fall under this definition should be regarded as medicinal products, subject to the applicable regulatory requirements for biologics, including vaccines and blood/plasma-based products. To obtain permission for clinical use, these must undergo rigorous evaluations for quality, safety, and efficacy at the pre-registration stage [51].

A somewhat different situation has arisen in the regulation of probiotic-based biologically active supplements or food ingredients. Because of the long-term historical use of various microbial strains in food products, these strains are considered inherently safe. The concept of safety for probiotics that have not undergone approval before entering the market in the United States is enshrined in the Generally Recognized as Safe (GRAS) program by the FDA [42,52] and in the Qualified Presumption of Safety (QPS) program by the EFSA in the EU (Table 4) [53].

This safety concept is based on the fact that strains previously used as ingredients in food products or dietary supplements have a long history of use in large human populations without any significant negative safety consequences [30]. From the perspective of food product legislation, the target group is always a healthy population, regardless of the historical use of the strain. When such a strain is an active ingredient in a medicinal product for the prevention or treatment of a disease in humans, evidence of efficacy and safety in the target population is required.

If a probiotic is marketed as a dietary supplement, it is regulated by the Dietary Supplement Health and Education Act (DSHEA). Although pharmaceuticals do not require pre-market approval, manufacturers are responsible for ensuring product safety and accurate labeling. According to the DSHEA, dietary supplements used since 1994 are permitted for inclusion in food products as ingredients [54]. If a probiotic is presented as a new dietary supplement or an ingredient not previously used, information about it must be submitted to the FDA through the New Dietary Ingredient (NDI) notification system [55].

Among the regulations considered, the European EFSA rules are the strictest. According to the EFSA guidelines (EU Regulation (EC) No 1924/2006), probiotics cannot be associated with health claims in the European Union unless they are supported by substantial clinical evidence (European Commission, 2006) [56]. In the EU, these claims must receive approval before being marketed. EU health claim legislation prioritizes consumer protection, though the specifics of regulation have differed between countries [57,58]. However, there are ongoing efforts to harmonize these regulations across the EU [57]. In contrast to the EU regulatory framework, the United States generally does not require pre-market approval for health claims, particularly for structure/function claims [57,58].

The majority of probiotic-related health claim applications submitted to EFSA have been rejected due to insufficient evidence of a cause–effect relationship between microorganism consumption and the claimed health benefit, as well as insufficient strain characterization and study design [59]. This fact rather reflects EFSA’s strict requirements to demonstrate a clear causal relationship and reproducibility of results in populations with widely different microbiomes. This restriction leads to cautious labeling and limited marketing [39,60].

To date, of the more than 400 health claims evaluated under Article 13(1) of Regulation (EC) No 1924/2006, only one has been authorized (European Commission, 2006) [56]: the claim that live yogurt cultures improve lactose digestion, attributable to the starter microorganisms *Lactobacillus delbrueckii* subsp. *bulgaricus* and *Streptococcus thermophilus*, provided they are present at a minimum level of 10^8^ colony-forming units (CFU)/g, as specified in Codex Alimentarius Standard No. 243/2003 [58]. All other health claims for probiotics that were submitted were either rejected or withdrawn, mainly due to inadequate characterization. However, EFSA’s scientific evaluation of health claims is required to adhere to the highest standards (European Commission, 2006) [56].

In the European Union, products containing probiotics generally do not require pre-market approval unless they are considered novel foods or contain specific health claims. In this case, the EFSA plays a leading role in evaluating the safety of probiotics and their use in food products. It should be noted that if a strain has not been used in the EU before May 1997 (QPS), it is considered a new food product, and pre-market approval is required in accordance with Regulation 2015/2283 on novel foods [46].

Thus, the regulatory framework strictly defines the depth and methodology of probiotic evaluation. The relationship between this framework and the safety and efficacy assessment of new probiotic products is governed by the intended use of the product and its history of safe use. The ultimate goal of such requirements is the complete safety of the probiotic for humans. Regulatory challenges affecting the commercialization of probiotics include the lack of harmonization of regulatory requirements, which can vary by region (e.g., as with drugs or dietary supplements), leading to confusion in interpreting requirements. Efficacy and safety standards also vary, increasing costs and complicating worldwide regulatory compliance, which in turn leads to differences in regional market access [29,61].

Taken together, current probiotic regulations reflect a tension between patient protection and innovation. While strict standards protect public health, rigid classification and international fragmentation can hinder adaptive, risk-based approaches tailored to strain biology and intended use. Deeper international harmonization and proportionate, genomics-based regulation could reduce duplication and support responsible innovation while maintaining safety oversight. Achieving this balance is essential for the sustainable global integration of probiotic and live biotherapeutic technologies.

### 2.2. Regulatory Framework of the Russian Federation

In the Russian Federation, the regulatory framework for probiotics, when positioned as dietary supplements, is governed by a number of laws, decrees, and regulations, regardless of how historically the probiotic has proven itself in terms of safety (Table 4). The circulation of probiotics in the form of dietary supplements is regulated by the Federal Law No. 52-FZ dated March 30, 1999, which addresses issues of sanitary and epidemiological surveillance and ensures food product safety; the Federal Law No. 29-FZ dated January 2, 2000, which sets out the general principles for regulating food products; and the Technical Regulations of the Customs Union TR TS 021/2011 and TR TS 029/2012 (Table 4), which contain requirements for the safety of food products, including dietary supplements; and the Government Resolution No. 982 dated December 1, 2009, which regulates the procedure for state registration of dietary supplements. An expert opinion must be provided to obtain a state registration certificate based on the results of expertise and testing. Rospotrebnadzor (Federal Service for the Oversight of Consumer Protection and Welfare of the Russian Federation) performs the functions of control, registration, and entering information into the Unified Register of Certificates.

Therefore, registration as a dietary or food supplement, as a specialized food product, is intended solely to supplement the daily diet and has no medicinal properties. This approach is similar to the international experience.

The state registration of probiotics as medicinal products is carried out by the federal executive authority, represented by the Ministry of Health of the Russian Federation, based on Federal Law No. 61-FZ (Table 4). This group of medicinal products also falls under the requirements of the Ministry of Health Order No. 403n dated July 11, 2017, “On the Approval of the Rules for the Sale of Medicinal Products for Medical Use, Including Immunobiological Medicinal Products, by Pharmaceutical Organizations and Individual Entrepreneurs Holding Licenses for Pharmaceutical Activities.”

The probiotic must meet the definition and requirements of the pharmacopoeia article concerning the production and formulation of the strain’s biomass, as well as the quality of the medicinal product for a specific dosage form, by the time of registration as a medicinal product [62]. In this context, the probiotic is defined as an immunobiological medicinal product that contains live or inactivated apathogenic microorganisms with antagonistic activity against pathogenic and conditionally pathogenic bacteria. The pharmacopoeia article also provides definitions for different groups of probiotics. It is also noted that probiotics are intended as medicinal products for the treatment and prevention of gastrointestinal diseases and the correction of dysbiosis of various etiologies.

During the registration process, a probiotic must be assigned to a specific pharmacotherapeutic group and be classified according to the Anatomical Therapeutic Chemical Classification. In the Russian Federation, probiotics registered as medicinal products have the following ATC codes: Saccharomyces boulardii (A07FA02), antidiarrheal microorganisms (A07FA), Lactobacillus (G01AX14), microorganisms producing lactic acid (A07FA01), and microorganisms producing lactic acid in combination with other drugs (A07FA51) [6].

In the Russian Federation, probiotics are regulated either as food supplements or medicinal products, with separate legal frameworks and regulatory bodies for each category (Table 4). Overall, despite the uneven regulatory environment both within Russia and internationally, there is broad consensus that probiotics (regardless of category) should meet scientifically sound standards of safety, quality, and efficacy.

According to legal requirements, the registration of a probiotic as a medical product must be based on preclinical and clinical studies using modern scientific methods that confirm its efficacy and safety for humans. From a scientific point of view, to prove the safety of a probiotic, it is also necessary to carry out procedures for identifying strains with characteristics of genomic safety, antimicrobial resistance, as well as genes for virulence, efficacy, and viability. Currently, the regulatory system’s understanding of probiotics is closer to the FDA’s position. In this regard, the Russian Federation is no exception, since even within the European Union, the regulatory framework for probiotics is not homogenous [27].

## 3. Safety, Toxicity and Other Risks Associated with Probiotics

Although probiotics are well known for their positive health effects and information about them often focuses on their beneficial impact, the potential risks they may carry should not be ignored. Several scientific publications highlight various health threats associated with the consumption of probiotics, prompting the need for more thorough investigation into their use and further research.

Under the current regulatory framework, probiotic safety assessment is based on a strain-specific risk assessment system that combines biohazard identification with an assessment of the context of use. Although probiotics are generally safe for healthy individuals, rare but clinically significant infectious events, including bacteremia and endocarditis, have been reported, particularly in patients with implanted devices, impaired barrier function, or severe comorbidities [63]. Additional risks arise from strain-specific metabolic activity, such as the production of D-lactate or biogenic amines, which justifies targeted biochemical screening during the development phase [64,65]. The identification of mobile antimicrobial resistance genes and their potential impact on intestinal resistance also requires whole-genome sequencing and detailed characterization of antibiotic resistance genes [42,66,67,68]. Therefore, regulatory frameworks such as the EFSA qualified presumption of safety and the FDA guidance on live biotherapeutics use an evidence-based approach that combines genomic analysis, phenotypic testing, manufacturing controls, and the clinical context to ensure product safety [31,35,59].

Probiotic safety assessment should be viewed as a hierarchical, multi-level framework rather than a collection of isolated observations. Foundational requirements include strain-level identification, phenotypic antimicrobial susceptibility testing, and whole-genome sequencing. Functional in vitro assays and preclinical in vivo models represent the intermediate level, while clinical trials and meta-analyses provide the highest level of human safety evidence, despite frequent design heterogeneity. Emerging computational tools serve as supportive rather than regulatory-accepted evidence. This hierarchy clarifies the relative weight and maturity of safety assessment methods.

Risk analysis of each probiotic strain can be a resource-intensive process. Although a low level of risk may be acceptable, as is the case for species like Lactobacillus and Bifidobacterium, establishing a balance between risk and potential benefit is important [4,19,69,70]. Reliable data on the efficacy and safety of probiotics are essential for this. Moreover, abroad, probiotics are researched across a broader range of diseases than in the Russian Federation, and this circumstance is another factor for a more comprehensive safety analysis of probiotics based on the results of already conducted clinical observations.

Probiotics and LBPs are generally well tolerated by healthy adult individuals [71]. Randomized studies involving up to 25 billion colony-forming units in healthy subjects did not show an increase in adverse events or laboratory abnormalities [72]. In contrast, studies involving mixed or high-risk populations, including patients receiving parenteral nutrition, those in intensive care, or patients with cancer, describe rare but serious adverse events in detail [73]. For example, one report on observational results described 93 cases of serious infections (with a 15% mortality rate), with fungemia (37.6% of cases), sepsis (31.2%), bacteremia (20.4%), endocarditis, and abscesses [74,75].

Not all safety data carry equal regulatory weight. Randomized controlled trials and robust meta-analyses provide the strongest clinical evidence for live biotherapeutics, whereas observational studies and case reports are primarily hypothesis-generating. Genomic screening and phenotypic susceptibility testing are increasingly required by regulators, while advanced computational models remain investigational. Distinguishing validated regulatory tools from emerging methodologies is essential to avoid overinterpretation of preliminary findings.

The analysis of the cited literary sources revealed heterogeneity in the initial parameters of the clinical observations described above. Differences were found in both study design and population type. The design description clearly noted 2 randomized controlled trials, 7 systematic reviews (including case reports), and 1 case report separately. The types of study populations were also heterogeneous. Three of the above studies were conducted in healthy or general adult populations. Four studies focused on high-risk patients, patients with poor health, cancer patients or intensive care unit patients. Three studies were conducted in mixed populations (healthy, compromised, seriously ill, or a wide range of age/risk groups). No strain specificity as such has been described. Observations were made when studying various Lactobacillus, Bifidobacterium, Saccharomyces, Streptococcus, Enterococcus, Bacillus, as well as their mixtures or yogurt/dairy.

No risk factors were identified in healthy adults, and no adverse events were observed at any dose. Randomized controlled trials reported no increased risk in the general or mixed populations, but rare serious adverse events were reported. Overall, adverse events and risk factors were rare or absent in healthy adults but were increased in populations with compromised health, critical illness, or immunodeficiency. Safety concerns and the need for caution were most frequently noted in the high-risk groups. Age over 60 years, infections such as *Clostridioides difficile*, antibiotic exposure, immunosuppression, and the use of *Saccharomyces* or *Lactobacillus* (especially *L. rhamnosus*) all appear to increase the risk [74]. In general, LBPs based on Lactobacillus and Bifidobacterium are safe for patients receiving enteral or parenteral nutrition [76].

Despite the large number of clinical observations regarding the use of probiotics, accurate data on the incidence of infections associated with their use remain unknown [77]. This is due to the lack of systematic reporting on adverse effects during clinical trials and the inefficiency of existing mechanisms for detecting harm after probiotic product registration [78]. At the same time, the results of published observations support the concept that clinically significant adverse effects of probiotics can be classified into several categories, including pathogenicity, infectivity, and virulence factors, such as toxicity, metabolic activity profile, and the specific properties of the microorganisms themselves [79]. In addition, probiotic microorganisms should not produce harmful substances during their metabolic activity [79].

Interpretation of clinical safety data is complicated by structural limitations in probiotic trial design. Substantial heterogeneity exists in endpoints—ranging from symptom-based outcomes to microbiome or biomarker measures—many of which lack regulatory validation. Interindividual variability, inconsistent strain identification, variable CFU dosing, and formulation differences further reduce reproducibility and comparability. Safety is often a secondary endpoint, limiting power to detect rare adverse events and contributing to inconsistent meta-analytic findings.

Based on clinical observation results, the most severe infection manifestations after probiotic use occur in patients with compromised immunity. This finding is related to the disruption of the balance between the symbiotic gut microbiota and the immune system [80]. In such vulnerable patient groups, intestinal barrier dysfunction increases the risk of adverse effects from probiotics [81] and may contribute to infection development, which can subsequently lead to sepsis or inflammation [19]. The use of *S. boulardii* and *L. rhamnosus* in such patients is not prohibited, despite some cases of infection, as numerous studies have proven their overall safety and effectiveness for humans [82].

In addition to increasing the risk of infection, probiotics may contribute to the development of a nonspecific immune response in humans [83,84]. One explanation for this phenomenon may be that the cell walls of Gram-positive bacteria contain peptidoglycan-polysaccharide complexes, which can activate immune cells [85]. Recent studies have linked probiotic-induced cytokine production, such as interleukins IL-1β, IL-6, tumor necrosis factor TNF-α, and interferons, to excessive immune reactions that can lead to autoimmune disorders or inflammation [12,86]. The potential use of probiotics in the treatment of autoimmune diseases is currently being actively studied. However, probiotic supplementation can prevent or cure autoimmune diseases or reduce cytokine levels [87].

The theoretical assumption that excessive stimulation of the immune system through probiotics could lead to an autoimmune response resulting in inflammation has not been confirmed [75]. Therefore, caution is required when using probiotics in autoimmune diseases, particularly in individuals at higher risk [88].

Despite the increasing interest in probiotics and prebiotics the prevention and treatment of allergic diseases, including atopic dermatitis, asthma, rhinitis, and food allergy, research results remain inconclusive or contradictory [87]. Although in vitro and in vivo studies suggest potential benefits, clinical efficacy is inconsistent due to substantial interindividual variability in lifestyle, microbiome composition, health status, gender, and age [89,90,91]. Accordingly, probiotic efficacy appears to be strain- and population-specific, with some strains being effective in certain patient groups but not in others [92,93], and there is evidence that probiotics may increase the risk of sensitization in individuals predisposed to allergic diseases [94]. Food allergy research has focused largely on infants, where the development of the gut microbiota in early life—which is strongly influenced by breastfeeding—plays a critical role in immune maturation [95]. Disrupted microbial colonization in early childhood is associated with an increased risk of food allergy [96], but studies of the use of probiotics in cow’s milk allergy remain inconclusive due to heterogeneity in host health status and genetic background [97].

Another category of potential side effects of probiotics can be gastrointestinal manifestations related to the metabolic activity of the probiotic strain and, at times, the toxicity of its metabolites. Such disturbances in gastrointestinal activity include vomiting, cramps, bloating, nausea, diarrhea, thirst, and taste disturbances that occur with the use of probiotics [98,99]. According to the authors of these studies, a decrease in the intensity of such symptoms and an increase in tolerance are generally observed with prolonged use of probiotics and depend on the type of microorganism and the host’s health status.

For instance, *Lactobacillus* spp. is largely responsible for D-lactic acidosis development in patients with short bowel syndrome [100]. However, under normal conditions, only a small amount of D-lactic acid produced in the gastrointestinal tract is absorbed by the host, while other bacteria in the gut quickly consume lactic acid, converting it into, for example, butyrate [101].

Some probiotic microorganisms contain genes for bile salt hydrolase. Species such as *Bifidobacterium*, *Pediococcus*, and *Lactobacillus* secrete this hydrolase, which deconjugates primary bile acids and reduces cholesterol reabsorption in the host’s body [102]. However, excessive deconjugation or dehydroxylation via 7α-dehydroxylase can trigger the formation of toxic or mutagenic secondary bile acids, which in turn can suppress the normal gut microbiota, causing intestinal mucosa inflammation and diarrhea [103]. Furthermore, excessive bile acid accumulation can lead to colon cancer development [104].

Many probiotic microorganisms can exhibit proteolytic, lipolytic, and/or saccharolytic properties depending on their species and strain. While displaying probiotic properties, the bacteria do not exhibit harmful biochemical properties for the host organism, such as β-glucosidase, α-chymotrypsin, β-glucuronidase, and N-acetyl-β-glucosaminidase activities, which are associated with intestinal diseases and can promote the formation of carcinogens and tumor promoters [105].

Attempts to use the biochemical properties of probiotics for the treatment of diseases related to metabolic disorders, such as metabolic syndrome (typically accompanied by dyslipidemia, glucose homeostasis disruption, high blood pressure, liver and kidney dysfunction, as well as excess body weight, abdominal obesity, and/or insulin resistance) [106], which is often associated with an increased risk of cardiovascular diseases and type 2 diabetes [107], and insulin resistance of various origins, have not yielded definitive results, either in prevention or in treatment. Conclusions were not universally positive, and some studies even reported unexpected side effects [87,108].

However, the risk of adverse effects (AEs) from probiotics cannot be ignored. Moreover, analyses of clinical studies show that probiotics have a low incidence of AEs—comparable to or lower than in control groups—but serious risks can arise in vulnerable populations. For example, an analysis of 622 randomized clinical studies found no statistically significant increase in the relative risk (RR) for total AEs (gastrointestinal, infections), including serious ones, with short-term probiotic use versus controls [109]. Of all the studies included, only 387 reported specific AEs (e.g., fungemia, bacteremia) potentially linked to probiotics. The study noted that long-term effects remain largely unknown.

Another analysis of 46 datasets found that probiotics reduced relapse rates versus placebo, though heterogeneity and data overlap limit broad claims [110]. A meta-analysis of nine controlled trials patients with inflammatory bowel disease showed higher AE risk with probiotics/synbiotics [111]. However, the authors found no clear explanation for this due to variations in study design and regimens. In 10 randomized controlled trials (1630 patients) on bismuth-containing quadruple therapy for Helicobacter pylori, probiotics lowered AEs from 27.98% (controls) to 16.85% [112]. To accurately assess AE levels, experts emphasize clarifying strain-specific effects, dose–response relationships, and optimal regimens in trials for evidence-based guidance [112,113]. Others note that inadequate harm data plague many treatments, not just probiotics [20].

To summarize the risk profile of probiotic use, the following points can be made. Strain-specific metabolic parameters (e.g., D-lactate, biogenic amines, etc.) and the ability of some strains to carry virulence loci or mobile antimicrobial resistance genes make metabolic syndrome and associated host metabolic states important for assessing probiotic safety. Clinical outcomes vary widely because host genetics, baseline microbiota, diet, medications, and comorbidities modulate response and susceptibility, which explains the heterogeneous results across studies [15,114,115]. Vulnerable populations (newborns, immunocompromised individuals, and critically ill individuals) are at greater risk; therefore, assessing regulatory compliance requires a combination of biochemical and metabolomic studies, whole genome sequencing for virulence, antibiotic resistance genes and motility mapping [63,66,87]. It is also necessary to evaluate probiotics in view of their ambiguous impact on certain fairly large categories of the population, for example, as described for the effect on calcium, phosphorus and bone metabolism, magnesium status, lipid and blood profile in postmenopausal women [116,117,118,119,120].

The possible transfer of antibiotic resistance genes from probiotic microorganisms to commensal pathogens within the gut flora is another aspect that raises concerns about the safety of probiotics [121]. Bacterial antibiotic resistance can result from genetic mutations or from the acquisition of resistance through horizontal gene transfer from other strains [122].

Mobile genetic elements (plasmids, transposons, and integrative conjugative elements) mediate horizontal gene transfer in the intestine under favorable conditions (high cell density, antibiotic exposure and inflammation). In vitro and in model systems, experimental studies have shown plasmid conjugation between enterobacteria and Gram-positive recipients, including LAB [44,123]. Regulatory requirements for the characterization of antimicrobial resistance genes (AMR) in probiotics and biopharmaceuticals are set out in regulatory documents from regulatory bodies such as the EFSA, FDA, and FAO/WHO [34,124,125]. The primary goal of these documents is to ensure that beneficial microorganisms do not act as a source of resistance genes that could be transmitted to human pathogens.

Many strains of *Lactobacillus* have natural resistance to antibiotics such as aminoglycosides, while *Bifidobacterium* is resistant to gentamicin, streptomycin, and mupirocin [85]. Additionally, *Lactobacillus* strains have been found to carry resistance genes to antibiotics such as erythromycin, tetracycline, streptomycin, chloramphenicol, lincosamide, macrolides, and streptogramins [28]. This characteristic of microorganisms, which ensures antibiotic resistance, raises particular safety concerns. Such properties could potentially be transferred to other strains, species, or even genera of bacteria, including those that are potentially harmful to humans and animals, limiting the effectiveness of antibiotic treatments. This is one of the most serious issues related to probiotics. The scientific literature contains numerous studies on this topic [87,126].

When antimicrobial resistance is specific to a bacterial species, it is commonly referred to as “intrinsic resistance” and is present across all strains of that species. Intrinsic antimicrobial resistance is not considered a safety risk. Conversely, when a strain of a normally susceptible species becomes resistant to a given antimicrobial, it is considered “acquired resistance,” which requires further investigation. Acquired resistance can arise either through the addition of genes (e.g., by obtaining exogenous DNA) or through mutation of one’s own genes [127,128].

Although gene exchange among viable microorganisms is expected to occur in open ecosystems such as the gastrointestinal tract, intrinsic antimicrobial resistance is generally regarded as having a low potential of horizontal spread, whereas acquired resistance encoded by additional genes is considered to carry a substantially higher risk of lateral transfer [128,129]. However, resistance due to mutation of chromosomal genes is considered to pose a low risk of horizontal spread [128,129].

Horizontal gene transfer through mobile genetic elements can pose a significant threat to human health through the development of antimicrobial resistance (AMR) [66,130]. AMR has emerged as a serious and acute global health threat, leading to increased mortality worldwide [131]. To exclude such risks, regulatory authorities (EFSA, FDA) and experts explicitly require strain characterization, phenotypic determination of minimum inhibitory concentration (MIC), and whole genome sequencing (WGS) annotated against carefully curated databases of antibiotic resistance/virulence genes (e.g., CARD, ARG-ANNOT, ResFinder) to determine whether resistance is innate or potentially transmissible [33,124].

The absence of ARG identified by WGS does not imply the absence of resistance mechanisms in probiotic lactic acid bacteria, as intrinsic or non-canonical resistance determinants may remain undetected due to database and methodological limitations, highlighting the need for cautious interpretation of safety assessments based on genomic data [132,133,134].

Although whole-genome sequencing is central to safety assessment, its reliability depends on the completeness and quality of resistance gene databases. Intrinsic or uncharacterized resistance mechanisms may remain undetected. Therefore, genomic findings should be integrated with phenotypic minimum inhibitory concentration testing to confirm concordance between predicted and expressed resistance. An integrated genomic–phenotypic approach remains the most robust strategy for antimicrobial resistance risk assessment.

Given the properties of microorganisms described above, strains intended for use as starter cultures or probiotics must be tested for antibiotic resistance and, specifically, for their ability to carry antibiotic resistance genes.

However, to assess the potential risks associated with probiotic resistance to antibiotics, this characteristic is not unique to probiotic strains. For example, wild strains of *Lactobacillus* and *Bifidobacterium* also contain antibiotic resistance genes that are similar to those found in probiotic isolates [101]. Additionally, *Enterococcus faecium*, which has a long history of safe use in food products despite possessing antibiotic resistance properties, serves as another example [85]. Therefore, probiotic strains do not pose a greater risk in this regard than *Lactobacillus* and *Bifidobacterium*, which naturally occur in the human microbiome. However, it is crucial to critically study the genetics of antibiotic resistance in probiotics [101].

Summarizing the published data on the efficacy and safety of probiotics, it is clear that the results are ambiguous and often contradictory. Some findings are isolated, based on single clinical cases, such as sepsis development in patients with established intravenous catheters [74]. Some of the consequences of clinical probiotic use have been attributed to poorly planned clinical studies and systematic errors in the evaluation of results [135].

Based on the meta-analyses of clinical trial results, conflicting results may be due to differences in the bacterial species, composition and dose of probiotics used. For example, studies show a wide range of doses, ranging from 10^8^ to 10^11^ cells or CFU, and different forms of administration (cheese, yogurt and milk, or probiotic capsule) may also cause further differences [14,15]. Some studies involved only women, while others examined the effects of probiotics on men and women in varying proportions. The clinical implications of this issue are unclear [15,136]. In some studies, the duration of exposure (3–6 weeks) may have been insufficient to detect changes [14,15].

In addition to the safety factors mentioned earlier, factors such as CFU dose, strain viability, presentation form, and manufacturing quality must be carefully controlled to ensure probiotic safety. Higher CFU doses, while potentially beneficial for increased efficacy, may increase the likelihood of gastrointestinal upset or infections in susceptible populations [20]. Viability is also important for probiotic safety, as non-viable strains may not provide beneficial health effects and may cause harm if improperly stored or managed [137]. Administration form also influences probiotic survival: some formulations provide better protection from gastric acidity and ensure higher strain viability at the site of action [138]. Furthermore, adherence to manufacturing quality standards is essential to ensure the potency, stability, and purity of probiotic products, reducing the risk of contamination or incorrect dosing.

The bottom line is that the results regarding probiotic use described in the scientific literature are irregular and vary significantly due to differences in probiotic types, their doses, and treatment regimens across studies [87]. Individual differences in microbiomes further complicate the problem [87,139]. Therefore, healthcare professionals do not recommend the use of probiotics in weakened patients, particularly those in critical condition [135].

Another class of probiotics, NGPs (e.g., specifically commensal gut bacteria such as *Bacteroides*, *Clostridium*, and *Akkermansia*), require greater safety consideration than traditional probiotics because they lack a “safe use history” and have a more “ambiguous” potential to cause infectious diseases [140]. NGPs have been linked to more specialized functions, including butyrate production, mucin degradation, increased intestinal barrier integrity, and modulation of host metabolism and inflammation [141]. Therefore, a thorough characterization of the strain is essential, encompassing whole-genome sequencing and the analysis of virulence factors, antibiotic resistance genes, and transferable genetic elements [142]. Additionally, safety should be evaluated through animal models of relevant diseases and human clinical trials [143].

NGPs demonstrate the importance of distinguishing between products with an extensive history of safe use and those lacking long-term human data. Unlike traditional probiotics, NGPs often require pharmaceutical characterization, extensive toxicology studies, and carefully designed early-stage clinical trials. Their development highlights the shift from empirical probiotic use to the precision microbiome therapy paradigm, which requires proportionate, strain-specific regulatory oversight.

The issue will be resolved with the accumulation of research results on the efficacy and safety of probiotics, the development of which is becoming increasingly intensive. Although regulatory authorities do not impose specific requirements on the preclinical and clinical research procedures for probiotics as LBPs, as previously noted, the specifics of probiotic research are actively discussed in scientific publications.

**Figure 1:**
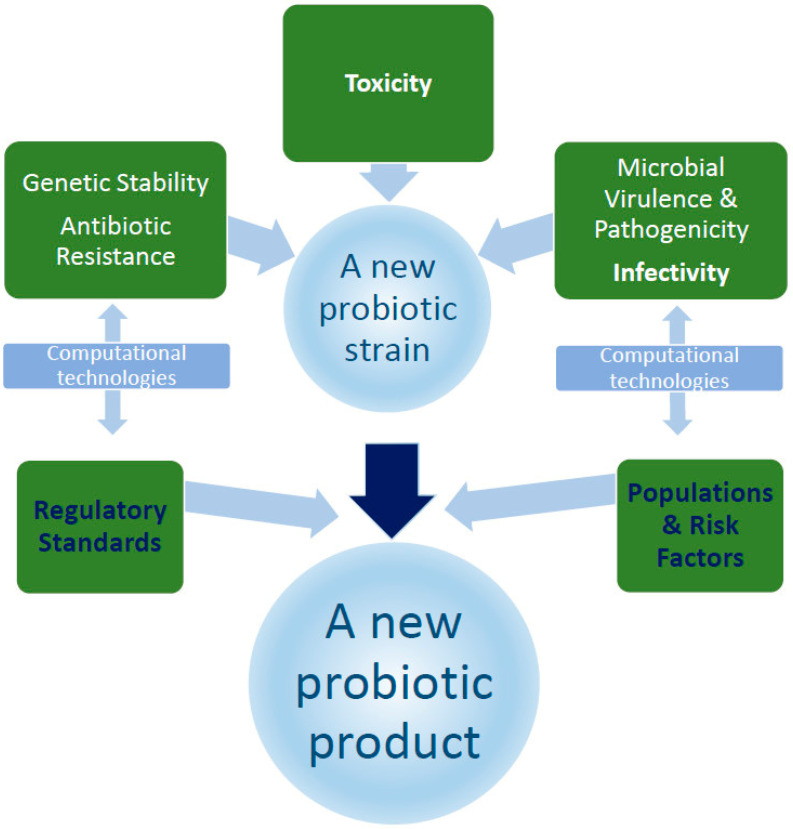
Key safety of new probiotics. Toxicity—evaluation includes testing for virulence factors, toxin production, hemolytic activity, and metabolic deleterious effects like D-lactate production; Microbial Virulence & Pathogenicity—assessing if the microbe has genes for invasion, colonization, or toxin production, even if it is a common gut bacterium; Infectivity—rare cases of infection (bacteremia, fungemia) have occurred, mostly in critically ill or immunocompromised patients; Genetic Stability—the microbe must remain genetically stable over time to ensure it does not evolve harmful traits during production or consumption; Antibiotic Resistance—checking for antibiotic resistance genes and the risk of them transferring to harmful gut bacteria. Probiotics should ideally be susceptible to clinically relevant antibiotics; Regulatory & Quality Standards—compliance of the probiotic with the requirements of “Generally Recognized As Safe” (GRAS) in the US or have “Qualified Presumption of Safety” (QPS) in the EU and safety is also tied to manufacturing standards (c-GMP); Populations and Risk Factors—this group of factors may include several points. Immunocompromised patients: Those with weakened immune systems (e.g., from chemotherapy or HIV) may be unable to clear a “live” probiotic, leading to infection. Critically Ill Patients: Probiotics are often contraindicated for patients in intensive care, those with severe acute pancreatitis, or those using central venous catheters. Infants: Premature infants or those with undeveloped immune functions require extreme caution due to potential microbial invasion and increased gut permeability; Computational technologies—possible areas of use for modern computer technologies (AI, big data, etc.).

Safety concerns for all new probiotics include the need to investigate the probiotic strain using genomic methods to identify potential virulence factors, antibiotic resistance genes, and other pathogenicity factors [82,144], metabolic activity [75], immune responses in terms of stimulation or suppression, especially in vulnerable populations such as children or patients with autoimmune diseases [19], and interactions with the host microbiome (Figure 1) [145]. In vitro tests should help identify antibiotic resistance, hemolytic activity, and other important strain parameters [145,146], whereas in vivo studies should be conducted using animal models with weakened immune systems to more accurately model potential risks in vulnerable populations [75,147]. Research should be conducted in accordance with the general Organization for Economic Cooperation and Development guidelines [144].

Standard in vitro functional and safety assays are widely used as preliminary criteria for selecting and characterizing probiotic candidates, including survival under conditions mimicking the gastric and biliary environment, adhesion to intestinal epithelial cells, antagonistic activity against pathogens, and absence of hemolytic activity [34]. While they do not prove in vivo efficacy, they provide key baseline data on function and safety, enabling the identification of priority strains for more in-depth studies [148]. Therefore, comprehensive reviews should explicitly address these fundamental assessments.

When planning probiotic safety and efficacy studies of probiotics, it is important to consider that properties depend on the specific strain. In this context, conducting preclinical safety evaluations for each individual probiotic strain before its use in humans is crucial [19,144]. An additional way to uncover the properties of a strain may be to develop mathematical models to predict the behavior and safety of probiotics in the gut [20,149].

New methods in the preclinical safety assessment of potential probiotic strains involve a multifaceted approach that goes beyond traditional methods and includes the use of modern computer technologies (Figure 1). Such studies are crucial to ensuring the safety and efficacy of new probiotic strains before they are used in humans, particularly in vulnerable populations [75,144].

**Figure 2:**
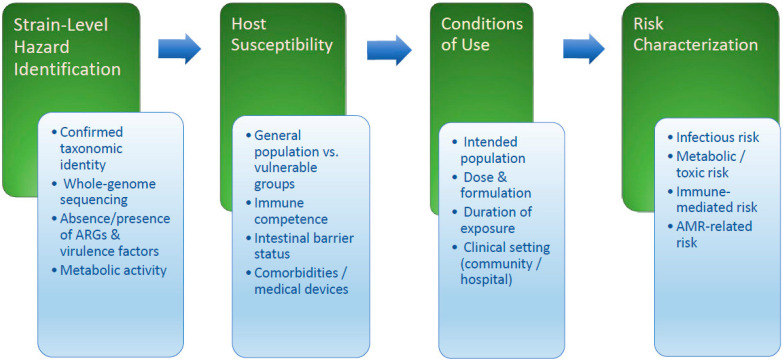
A brief flowchart of the regulation process for new probiotics.

When planning and conducting clinical trials of new probiotic strains, it is also necessary to adhere to the requirements of regulatory bodies, maintaining close collaboration with them (Figure 2) [50]. Additionally, it is important to consider the specific characteristics and properties of the probiotic strains [47,50,149], while ensuring the safety of all ingredients in the probiotic composition [47,125,150].

In summary, probiotic safety is a complex issue, dependent on various factors, such as strain-specific properties, dosage, viability, and delivery method. Although probiotics are generally safe for healthy individuals, they may cause infections in vulnerable populations, including those with weakened immune systems or compromised barriers. Strain-specific metabolic activity and the presence of antimicrobial resistance genes may also pose risks. A thorough safety assessment, including whole-genome sequencing, phenotypic testing, and preclinical studies, is crucial, especially for NGPs, which do not have a long safety history. A comprehensive approach and adherence to regulatory requirements are essential to ensure probiotic safety. Probiotic safety assessment requires an integrated, multi-tiered approach combining genomic, phenotypic, functional, and clinical evaluation. Safety should be interpreted as strain-specific and host-dependent rather than generalized at the genus or species level. Progress depends not only on advances in sequencing and bioinformatics but also on standardized methodologies, harmonized reporting, and validated biomarkers linking microbial mechanisms to clinical outcomes. Such integration is essential to balance innovation with patient protection in microbiome-based therapeutics.

## 4. Computational Technologies in the Development of New Probiotics

Probiotic research is increasingly using computational technologies, including whole-genome sequencing, in silico functional annotation, comparative genomic analysis, pathway reconstruction, and AI-based predictive models, to support the analysis and selection of specific strains [132,133,151]. These approaches are primarily used in the early stages of discovery and safety screening to identify potential antibiotic resistance genes, virulence-associated traits, and mobile genetic elements, enabling risk assessment and regulatory evaluation, and supporting phenotypic analyses and clinical validation [31,152,153].

For example, Senan et al., in 2015, showed that genomic screening using specialized databases can identify antibiotic resistance genes and virulence factors, saving time and covering safety aspects that are costly to cover using traditional clinical trial methods [16,154]. Genomic screening has been proposed as an effective and time-saving alternative or adjunct to standard safety protocols; however, specific time comparisons have not been provided. However, in the same year, Doron et al. (2015) conducted an analysis based solely on traditional literature review methods based on the results of 622 probiotic safety studies [63]. This suggests that as of 2015, the application of computational methods to probiotic safety assessment was in its early stages of integration.

Computational technologies have been incorporated across all stages of the probiotic research and development process, encompassing genomic screening, predictive safety evaluation, functional modeling, and regulatory assessment. Whole-genome sequencing-based genomic screening allows for the in silico detection of antibiotic resistance genes, virulence-associated factors, and mobile genetic elements, thus providing a basis for modern probiotic safety evaluation [20,133,155,156]. Toxicology predictions, including genome-based hazard forecasts and developing machine-learning models, are being researched to anticipate adverse characteristics and reduce dependence on extensive in vivo testing, despite their application to probiotics being at a preliminary stage [65,157]. Host–microbe interaction modeling, using systems biology, metabolic network reconstruction, and in silico simulations, provides insight into potential functional impacts and strain-specific mechanisms, supporting hypothesis generation before clinical validation [158]. Simultaneously, real-time monitoring technologies integrating bioinformatics with multi-omics and digital health data are starting to allow for the long-term tracking of probiotic strains and host responses, especially in translational and post-market settings [159,160].

Computational tools can be applied to mitigate risks associated with NGPs by enabling whole-genome sequence analysis, early detection of antibiotic resistance genes, virulence-related traits, the potential for horizontal gene transfer, and other potentially negative metabolic characteristics, thus speeding up strain selection and regulatory assessment [140,161]. In particular, these approaches are crucial for non-traditional probiotic candidates derived from gut commensals that lack a long history of safe use—such as *Akkermansia*, *Faecalibacterium*, *Bacteroides*, and *Clostridium* species—where in silico analyses complement phenotypic testing to accelerate safe development [153,162].

These computational tools are evolving and becoming integrated into regulatory processes over time, where genomic and bioinformatic analysis complement phenotypic testing to facilitate safety assessment and documentation, and highlight the need for standardized, transparent, and validated computational processes [163,164].

Recently, some modern technologies have been transforming safety protocols through a range of advances. Thus, genomic risk screening can be accelerated through rapid identification using artificial intelligence (AI) algorithms (in particular, DeepMicro, which analyzes sequencing data at high speed to identify bacterial species and potential pathogens with greater speed and accuracy than cultivation-based methods); identification of ARG and virulence detection (for example, using a big data platform such as CARD, a comprehensive database on antibiotic resistance, or ResFinder integrated with AI to predict potential resistance-related genes and virulence factors at the genomic level); and prediction based on a machine learning model with the identification of specific tRNAs that distinguish safe probiotics against dangerous intestinal pathogens, which helps to carry out screening at an early stage [156,165,166].

Probiotic research utilizes computational tools, including machine-learning-based predictors like DeepMicro and homology-based genomic screening platforms like CARD and ResFinder, which vary significantly in their mechanisms and interpretability. DeepMicro uses deep learning to infer results from genomic or microbiome data but relies heavily on high-quality training data and external validation. In contrast, CARD and ResFinder use carefully curated databases to identify known antibiotic resistance genes, providing clear results but may fail to detect novel, unusual, or modulated resistance mechanisms [67,152,167,168]. These approaches can result in false positives and false negatives due to factors such as annotation thresholds, database bias, fragmented assemblies, or model overfitting, highlighting the need for careful validation against phenotypic data and careful interpretation when assessing the safety and efficacy of probiotics [67,132,133].

At this point, it can be concluded that unresolved challenges in data quality, batch effects, annotation consistency, and pipeline standardization across computational tools, including both AI-based and homology-based ones, remain significant obstacles to performing robust, reproducible, and regulatory-interpretable probiotic safety assessments.

AI plays a major role in the development of predictive toxicology, in which microbial metabolites and their physiological functions can be predicted using a machine learning (ML) model, and potential toxic activity can be identified before in vivo testing [21,156]. The assessment of the degree of safety for vulnerable groups using AI is conducted by analyzing large amounts of data and identifying specific “risk factors” for side effects, such as impaired intestinal barrier function or immunocompromised conditions, which allows for safer use of probiotics [21,156]. The use of AI to analyze big data for real-time monitoring will simplify clinical trials by providing monitoring of adverse events and more accurate tracking of specific strains in real time to evaluate a more complete safety profile of probiotics [19].

AI can be used to analyze big data from various omics technologies to create a safety profile for probiotics based on, for example, metabolomics, where AI evaluates metabolite production to ensure that strains do not produce harmful substances associated with various health risks [156,164]. The growth of AI capabilities is fueling new fields, such as probiogenomics, where computational models are used to predict how a probiotic’s genome will interact with the host immune system or existing microbiota, ensuring stable colonization without side effects [164,169].

Computational tools such as whole genome sequencing and bioinformatics analysis are increasingly being included in probiotic regulatory dossiers as supporting evidence for strain characterization and safety, provided that the methods are transparent, reproducible, and interpretable alongside phenotypic data [31,132,133]. In contrast, AI-based predictions are currently considered experimental and must undergo rigorous external validation and biological confirmation before they can be considered by regulators, highlighting that computational methods complement existing safety assessments based on standardized phenotypic testing and strain-level documentation [151,153,167]. Although AI and deep learning models have potential in probiotic research, their acceptance by regulatory authorities is hampered by the lack of clarity in their performance, as uninterpretable predictions do not provide a clear link to biological processes and specific safety characteristics of strains [167,170].

To address this issue, the EU adopted the Artificial Intelligence Act, which sets out harmonized rules in this area and now requires disclosure to prevent the use of “black box” models, which do not reveal information about the decision-making process, in applications that pose a high security risk (Regulation (EU) 2024/1689) [171].

While AI undoubtedly speeds up screening, regulatory standards still require traditional human review. Therefore, the use of AI can raise several potential challenges. For example, the high variability of biological datasets on different platforms for sequence determination causes problems with the reliability of the source data, which undoubtedly underestimates the accuracy of the AI model [154,172]. Despite their potential, AI-based forecasts still require careful experimental and clinical verification to ensure human safety due to the lack of a unified validation system for methods [173]. Ethical and regulatory issues related to data privacy, algorithm transparency, and lack of coordinated guidance for AI-based human security research are major obstacles to technology development [174].

Based on the latest advances in digital technology, it can be assumed that in the near future it will be possible to observe the integration of genome-wide screening with traditional clinical validation representing an emerging approach rather than an established standard, with computational methods most applicable to early-stage strain selection and mechanistic risk identification prior to expensive clinical trials.

In summary, probiotic research is more frequently incorporating computational methods like whole-genome sequencing, bioinformatics, and advanced AI technologies to hasten the process of early-stage strain identification, safety evaluation, and risk assessment by identifying antibiotic resistance genes, virulence factors, and other possible hazards. Phenotypic testing is now often complemented by genomic and bioinformatic methods in regulatory assessments; however, AI-based predictions are still largely experimental due to difficulties with data quality, standardization, interpretability, and validation. Computational methods are anticipated to complement traditional clinical and safety evaluations, with their primary influence taking effect before in vivo testing and clinical trials.

## 5. Conclusions

Many physicians advocate the use of probiotics, as they are capable of treating, preventing, or alleviating the course of various diseases. However, despite their numerous positive health effects, the potential risks associated with their use must also be considered. Most known side effects have been described in individual clinical cases, and only a few randomized controlled trials have reported adverse reactions.

While the consumption of probiotics by healthy individuals is generally safe, their use in individuals with weakened immune systems requires caution. Different probiotic strains may have different effects on health, and their impact on disease conditions should not be assumed to be the same across strains. Clinical data show that although probiotics are generally safe for healthy populations, they may pose risks in vulnerable groups, underscoring the need for careful risk–benefit assessment. Advances in microbiome science and omics technologies have expanded understanding of their mechanisms of action, while also revealing safety risks that are highly strain-, dose-, host-, and context-dependent.

The regulations and guidelines for the registration of new probiotic products are continually being improved. The ultimate goal of these requirements is not only to demonstrate efficacy but also to ensure the safety of commercial probiotics, the range of which has significantly expanded with the growing market for probiotic products. Therefore, regulatory frameworks clearly distinguish between food-grade probiotics and medicinal products or live biotherapeutics, with the latter requiring rigorous evidence of safety, efficacy, and quality, including genomic characterization. Overall, future probiotic development progress will depend on harmonized regulation, robust clinical evidence, and the integration of modern genomic and computational tools into safety evaluation.

## Figures and Tables

**Table 1:** Types of products with probiotic activity.

Types of Products	Definition
Probiotics	Live microorganisms that, when administered in adequate amounts, confer a health benefit on the host (ISAPP). Product that contains live or inactivated apathogenic microorganisms with antagonistic activity against pathogenic and conditionally pathogenic bacteria
NGPs	Specifically developed for the treatment of specific diseases with targeted therapeutic effects, non-traditional probiotics. NGPs do not have a single official consensus definition from ISAPP.
Prebiotics	A substrate that is selectively utilized by host microorganisms conferring a health benefit (ISAPP).
Synbiotics	A mixture comprising live microorganisms and substrate(s) selectively utilized by host microorganisms that confers a health benefit on the host (ISAPP).
Postbiotics	Preparation of inanimate microorganisms and/or their components that confers a health benefit on the host (ISAPP).

Note: ISAPP—International Scientific Association for Probiotics and Prebiotics. All explanations are provided in the article’s text.

**Table 2:** Summary of the regulatory definitions of probiotic microorganisms.

Term	Definition	Regulatory Status	Primary Use Case
Probiotic	Live microorganisms which when administered in adequate amounts confer a health benefit on the host (FAO/WHO, ISAPP)	Food/Supplement	General health maintenance
GRAS (Generally Recognized as Safe)	An FDA classification of food substances that demonstrates they are reasonably safe under the conditions of their intended use.	FDA Safety Class	US food ingredient safety
QPS (Qualified Presumption of Safety)	The QPS approach assesses the taxonomic identity, body of relevant knowledge and safety of microorganisms intentionally added to the food and feed chain.	EFSA Safety Class	EU harmonized safety list
Novel Food	Novel Food is defined as food that had not been consumed to a significant degree by humans in the EU before 15 May 1997, when the first Regulation on novel food came into force	EU Food Category	Newly introduced microorganisms
Dietary supplement	It is defined as a product intended for oral administration that contains a “food ingredient” (such as vitamins, minerals, amino acids, or probiotics) to supplement the diet.	FDA: Regulated under the Dietary Supplement Health and Education Act (DSHEA) of 1994; EU: Regulated under Directive 2002/46/EC	For all supplements
LBP	- biological products that: (1) contain live organisms (bacteria or yeast); (2) are used to prevent, treat, or cure a disease; and (3) are not vaccines (FDA). - medicinal products containing live micro-organisms (bacteria or yeasts) for human use (Ph. Eur).	Biological Drug	Disease treatment or prevention

**Table 3:** Comparative Safety Requirements.

Authority	FDA	EFSA	Ph. Eur.
Regulatory Framework	GRAS/LBP	QPS/Novel Food	Medicinal Products
Key Safety Requirement	- The focus is on risks; probiotics are permitted to be used as food additives or dietary supplements without pre-market approval if they are generally recognized as safe for consumption (GRAS).	- Focuses on hazards; uses QPS list to simplify safety checks of known species.	- Sets pharmacopoeial standards (e.g., Issue 12.3) for purity, potency, and identity of LBPs.
Approach to Claims	- More flexible; allows structure/function claims for supplements.	- Strictest globally. “Probiotic” is considered a health claim and is generally not permitted on labels	- The focus is on technical compliance with the requirements for pharmaceutical-grade strains.

**Table 4:** Legal regulatory framework for probiotic products in the United States (US), the European Union (EU) and the Russian Federation (RF).

Probiotic Products	US	EU	RF
Traditional probiotics	- Generally Recognized as Safe (GRAS) - Dietary Supplement Health and Education Act (DSHEA) (FDA)	- Qualified Presumption of Safety (QPS) - Regulation (EU) 2015/2283 of the European Parliament [46] (EFSA)	- Federal Law No. 52-FZ “On Sanitary and Epidemiological Welfare of the Population” - Federal Law No. 29-FZ “On the Quality and Safety of Food Products” - Technical Regulations of the Customs Union TR TS 021/2011 “On Food Safety” and TR TS 029/2012, contain requirements for the safety of food products (Rospotrebnadzor)
LBPs	- Early Clinical Trials with LBP, 2016 [36] (FDA)	- Directive 2001/83/EC - EDQM - European Pharmacopoeia Commission et al. 3053E General monograph (EMA)	Federal Law No. 61-FZ “On Circulation of Medicines.” (Ministry of Health)
NGPs	- Early Clinical Trials with LBP, 2016 [36] (FDA)	- Directive 2001/83/EC - EDQM - European Pharmacopoeia Commission et al. 3053E General monograph (EMA)	Federal Law No. 61-FZ “On Circulation of Medicines.” (Ministry of Health)

Note: All explanations are provided in the article’s text.

## Data Availability

The original contributions presented in this study are included in the article. Further inquiries can be directed to the corresponding author.
